# Deep Neural Network Augments Performance of Junior Residents in Diagnosing COVID-19 Pneumonia on Chest Radiographs

**DOI:** 10.3390/diagnostics13081397

**Published:** 2023-04-12

**Authors:** Yangqin Feng, Jordan Sim Zheng Ting, Xinxing Xu, Chew Bee Kun, Edward Ong Tien En, Hendra Irawan Tan Wee Jun, Yonghan Ting, Xiaofeng Lei, Wen-Xiang Chen, Yan Wang, Shaohua Li, Yingnan Cui, Zizhou Wang, Liangli Zhen, Yong Liu, Rick Siow Mong Goh, Cher Heng Tan

**Affiliations:** 1 Institute of High Performance Computing (IHPC), Agency for Science, Technology and Research (A*STAR), 1 Fusionopolis Way, #16-16 Connexis, Singapore 138632, Singapore; 2 Department of Diagnostic Radiology, Tan Tock Seng Hospital, 11, Jalan Tan Tock Seng, Singapore 308433, Singapore; 3 Lee Kong Chian School of Medicine, 11, Mandalay Road, Singapore 308232, Singapore

**Keywords:** COVID-19, chest X-rays, deep neural networks, AI assistant for diagnosing

## Abstract

Chest X-rays (CXRs) are essential in the preliminary radiographic assessment of patients affected by COVID-19. Junior residents, as the first point-of-contact in the diagnostic process, are expected to interpret these CXRs accurately. We aimed to assess the effectiveness of a deep neural network in distinguishing COVID-19 from other types of pneumonia, and to determine its potential contribution to improving the diagnostic precision of less experienced residents. A total of 5051 CXRs were utilized to develop and assess an artificial intelligence (AI) model capable of performing three-class classification, namely non-pneumonia, non-COVID-19 pneumonia, and COVID-19 pneumonia. Additionally, an external dataset comprising 500 distinct CXRs was examined by three junior residents with differing levels of training. The CXRs were evaluated both with and without AI assistance. The AI model demonstrated impressive performance, with an Area under the ROC Curve (AUC) of 0.9518 on the internal test set and 0.8594 on the external test set, which improves the AUC score of the current state-of-the-art algorithms by 1.25% and 4.26%, respectively. When assisted by the AI model, the performance of the junior residents improved in a manner that was inversely proportional to their level of training. Among the three junior residents, two showed significant improvement with the assistance of AI. This research highlights the novel development of an AI model for three-class CXR classification and its potential to augment junior residents’ diagnostic accuracy, with validation on external data to demonstrate real-world applicability. In practical use, the AI model effectively supported junior residents in interpreting CXRs, boosting their confidence in diagnosis. While the AI model improved junior residents’ performance, a decline in performance was observed on the external test compared to the internal test set. This suggests a domain shift between the patient dataset and the external dataset, highlighting the need for future research on test-time training domain adaptation to address this issue.

## 1. Introduction

The Severe Acute Respiratory Syndrome Coronavirus 2 (SARS-CoV-2) outbreak [1], initially detected in Wuhan, Hubei, China, has rapidly escalated into a worldwide pandemic [2]. The National Centre for Infectious Diseases (NCID) [3] has been at the forefront of Singapore’s COVID-19 response. As of the time of writing, the Ministry of Health (MOH) has recorded over 2.2 million confirmed cases in Singapore [4].

Patients with COVID-19 pneumonia often exhibit similar symptoms to other viral diseases, such as Middle East Respiratory Syndrome [5], and their imaging findings are often non-specific, presenting a diagnostic challenge [6,7,8]. Currently, the definitive method for diagnosing COVID-19 infection is the reverse transcriptase polymerase chain reaction (RT-PCR) [9]; however, limitations in diagnostic testing resources can hinder its accuracy [10,11,12]. In this context, chest imaging techniques [13], including computed tomography (CT) and chest radiography (CXR), are essential in the context of patient triaging and making treatment decisions [12,14]. Despite being less sensitive than CT, CXR is more widely adopted as it is faster, exposes patients to lower levels of radiation, and is potentially more cost-effective [7,15,16]. Therefore, junior residents, who are often the first point of contact, are expected to interpret CXRs of COVID-19 patients in many institutions.

In recent years, the application of deep learning models to clinical problems has shown significant potential in facilitating auto-diagnosis of diseases and providing real-time procedural support, particularly in the field of healthcare [17,18,19]. Several studies have explored the use of deep learning models for diagnosing COVID-19 through analysis of chest X-rays [20,21]. For instance, Jordan et al. collected a CXR dataset from NCID to train an AI model with the DenseNet as backbone to detect COVID-19 pneumonia. In response to the rapidly evolving global pandemic during COVID-19, they quickly deployed the trained model to NCID [22]. Abul et al. adopted a convolutional neural network (CNN) to extract feature representations from CXRs, and connected it with various classifiers, such as support vector machine (SVM), pattern recognition network (PRN), decision tree (DT), random forest (RF), and k-nearest neighbours (KNN), to perform COVID-19 detection [23]. Linda et al. introduced a novel COVID-Net for diagnosing COVID-19 from CXRs in publicly available datasets [24]. This approach incorporates an explainability method to not only provide clinicians with a deeper understanding of the critical factors associated with COVID-19 cases and improved screening, but also to enhance the transparency and accountability of COVID-Net by ensuring that its decisions are based on relevant information extracted from CXR images. However, the diagnostic performance of these models has yet to be validated in a clinical setting due to the limited size of CXR datasets used for training [24,25,26]. Moreover, it has been reported that lab-trained models may experience a significant decline in performance when deployed in clinical practice [27].

The motivation of this research is to harness AI’s potential to improve the diagnostic process, optimize healthcare resources, and ultimately enhance patient care. Specifically, we develop a deep neural network to differentiate COVID-19 from other forms of pneumonia and explore the potential of AI techniques to enhance the diagnostic accuracy of junior residents, who often serve as the primary point-of-contact in the diagnostic process. In order to achieve the objectives of this study, an interdisciplinary approach has been adopted that combines clinical research, image diagnostics, and AI models. The primary aim of the study is to collect structured data, including CXRs and RT-PCR results, and use these data to develop a three-class classification AI model that can accurately distinguish between non-pneumonia, non-COVID-19 pneumonia, and COVID-19 pneumonia cases. Subsequently, the study seeks to assess the effectiveness of the AI model in enhancing the diagnostic precision of novice residents by implementing and evaluating its performance. Specifically, we collected a patient dataset and used it to train and validate the AI model. Then, the trained AI model has been deployed in the Tan Tock Seng Hospital (TTSH), where its effectiveness in improving junior residents’ diagnostic accuracy has been evaluated. In addition, the study has investigated the performance of junior residents with different levels of training, both with and without the assistance of the deployed AI model.

## 2. Materials and Methods

### 2.1. Dataset

The dataset utilized in this work comprised a total of 5051 CXRs obtained from the NCID Screening Centre and Tan Tock Seng Hospital (TTSH) in Singapore. CXRs that were conducted between February 2020 and early April 2020 were included in the dataset. Two senior radiologists, each with over 15 years of experience, annotated the class label of the CXRs as pneumonia or non-pneumonia and used them as the reference standard. All CXRs were reviewed independently by senior radiologists who were unaware of any clinical details.

Patients were classified into three groups based on their clinical presentation and diagnostic results. Patients were classified as positive for COVID-19 pneumonia if they tested positive on the PCR test and had a CXR positive for pneumonia. Patients were classified as having non-COVID-19 pneumonia if their CXR was positive for pneumonia, but they tested negative on the PCR test. The remaining patients were grouped to the non-pneumonia class.

The dataset contained a total of 607 COVID-19 pneumonia cases, 570 cases of non-COVID-19 pneumonia (including viral, bacterial, and fungal pneumonia), and 3874 non-pneumonia cases. To form the training, validation, and test sets, CXRs were chosen randomly from each category within the dataset. These three sets comprised 70%, 10%, and 20% of the total data, respectively. Table 1 provides a summary of the dataset split statistics.

### 2.2. External Test Set

In order to assess the potential of AI assistance to improve the diagnostic accuracy of junior residents, a separate dataset consisting of 500 CXRs were used. This external test set was reviewed by three junior residents with varying levels of training, both with and without the assistance of the developed AI model.

The external dataset was also obtained from the same institution but was collected during a different time period. It included 72 cases of COVID-19 pneumonia, 49 cases of non-COVID-19 pneumonia, and 379 cases of non-pneumonia, as detailed in Table 1.

### 2.3. Neural Network Architecture and Training Strategy

**Figure 1:**
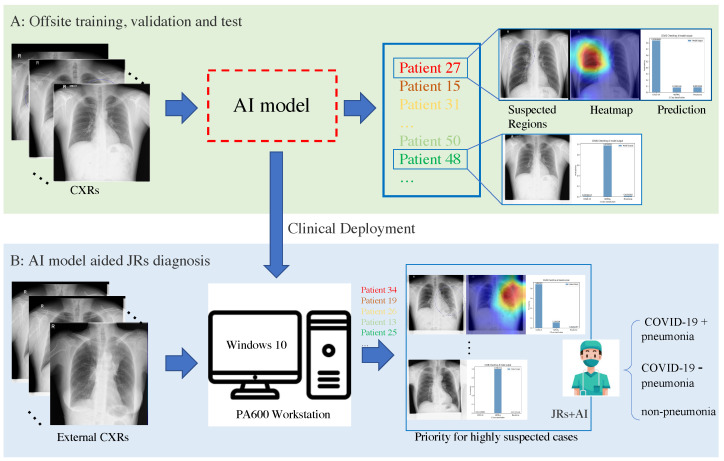
The workflow for training the AI model and conducting AI-aided diagnosis. Stage (**A**) involves training, validating, and testing the AI model for diagnosing CXRs. In Stage (**B**), the trained AI model is deployed in the hospital, and the junior residents (JRs) can upload CXRs to obtain the predictions and heatmaps generated by the AI model. By using AI results, the JRs can make more informed decisions.

The workflow of the AI model-aided diagnosis is presented in Figure 1. Initially, we developed an end-to-end CNN-based framework [28] to classify CXRs as non-pneumonia, non-COVID-19 pneumonia, or COVID-19 pneumonia. In the second stage, three junior residents, each with varying levels of training, were enlisted to review the external test set both with and without AI assistance. During the AI-assisted review, the junior residents were provided with the probability output and relevant heatmap for each case, generated by the trained model.

**Figure 2:**
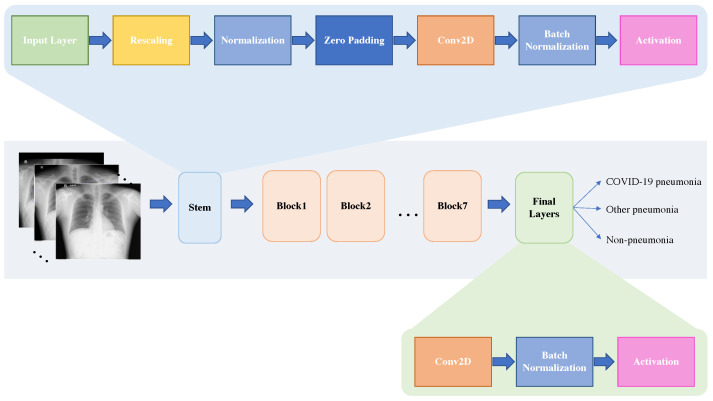
The architecture of EffcientNet-b7 for CXRs classification. The model takes CXRs as inputs and ultimately classifies them into three categories: non-pneumonia, non-COVID-19 pneumonia, and COVID-19 pneumonia.

To extract the features of the input CXRs, we employed an EffcientNet-b7 [29] model as the backbone, replacing the output layer with one that includes three neurons with the softmax activation function to output the final predictions. The architecture of the EfficientNet-b7, comprising Block1 to Block7, Stem, and Final Layers, is illustrated in Figure 2. Given the imbalanced patient dataset, we minimized the weighted cross-entropy loss (WCEL) to optimize the AI model as follows:

(1) $$L=-\sum_{i=1}^{N}\sum_{j=1}^{C}w_{j}y_{ij}\log\left(p_{ij}\right),$$

where *N* is the number of samples in the dataset, *C* is the number of classes, $w_{j}$ is the weight assigned to class *j*, $y_{ij}$ is the true label for class *j* of sample *i*, and $p_{ij}$ is the predicted probability of sample *i* belonging to class *j* obtained from the output of the model. In this work, the weight for each class is $w_{j}=\frac{N}{N_{j}}$ with $N_{j}$ as the number of samples in class *j*.

We trained, validated, and tested our AI model on the internal dataset, choosing the model that displayed the top AUC score during validation to generate probability scores and heatmaps for use in our experiment.

### 2.4. AI Model Deployment & Diagnosis

In this study, the trained model was executed on a virtual machine running Ubuntu 18.04, which was hosted on a Windows 10 workstation equipped with two Nvidia 2080Ti GPUs, located within the Department of Diagnostic Radiology. In adherence to the hospital’s security guidelines, formal authorization was secured from the Integrated Health Information Systems (IHiS) committee in Singapore, which is in charge of managing the IT risk and security for Tan Tock Seng Hospital.

To evaluate the impact of our AI model in enhancing the performance of junior residents, we recruited three junior residents with varying levels of training for the study. The residents independently reviewed the external dataset of 500 CXRs, initially without the aid of the AI model. After a deliberate 3-month hiatus, the same dataset of 500 CXRs was reviewed by all three junior residents, this time with the aid of AI in the form of probability outputs and relevant heatmaps.

## 3. Results

### 3.1. Verify on AI Model

The effectiveness of the developed AI model was verified by comparing it with five deep learning methods [6,30,31,32,33] designed for COVID-19 diagnosis using CT scans and CXRs. The performance of our method and the five peer methods for the test set and the external test set in terms of AUC scores (with the macro-averaging strategy) and 95% confidence interval (95% CI) are reported in Table 2, which show that our model outperforms the other five methods significantly, achieving an AUC score of 0.9520 (95% CI: 0.9479–0.9585) for the internal test set and 0.8588 (95% CI: 0.8570–0.8623) for the external test set. Specifically, it improves the AUC score of the peer methods by 1.25% and 4.26% for the test set and the external test set, respectively. This evidence suggests that the proposed AI model demonstrates efficacy in diagnosing pneumonia. All the tested methods demonstrated significant performance degradation on the external test, potentially due to a data distribution mismatch between the TTSH dataset (training, validation, and test set) and the external test set.

**Figure 3:**
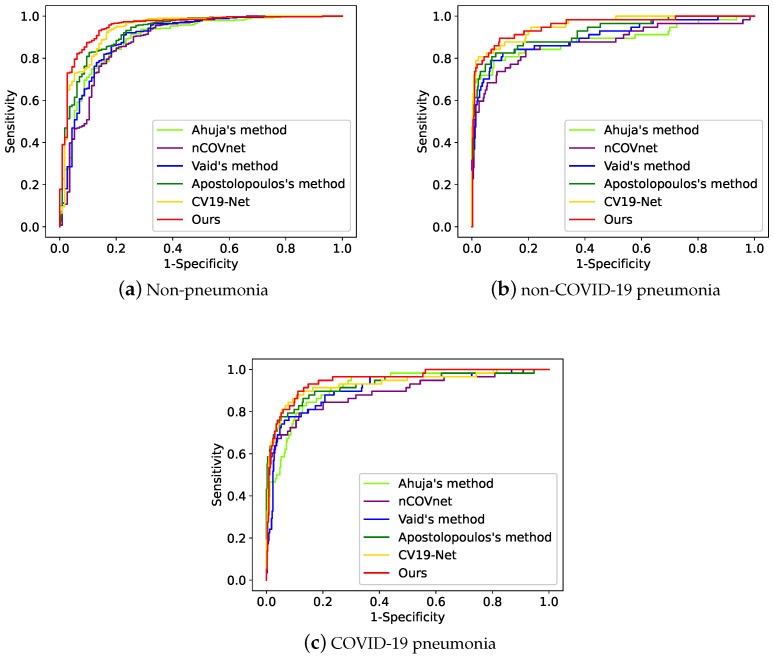
The ROC curve of the peer methods (i.e., Ahuja’s method [30], nCOVnet [31], Apostolopoulos’s method [33], and CV19-Net [6]) and ours on the three classes.

**Figure 4:**
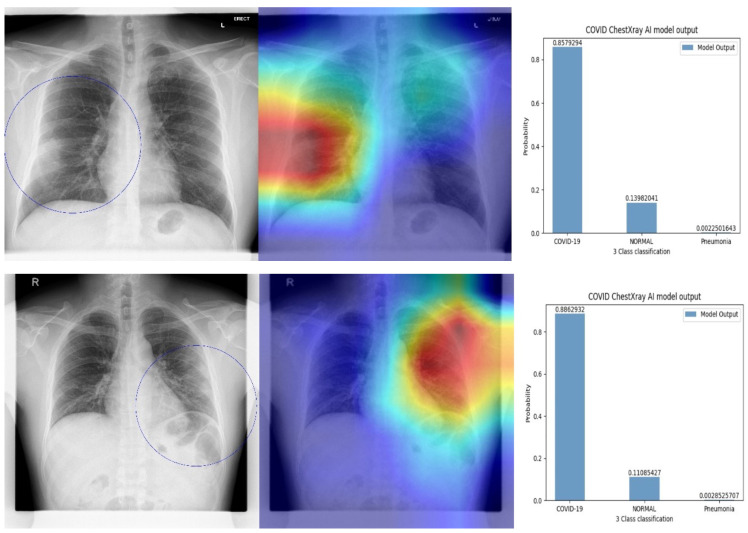
Examples of heatmaps and predictions generated by the AI model. The left-most image displays grayscale chest radiographs with superimposed blue circles indicating the suspicious area showing signs of possible COVID-19 infection based on the AI model’s heatmaps. The middle image shows the heatmaps overlaid on the original CXRs, with the highlights indicating the anatomical regions that contribute most to the final model predictions. The right-most image displays the predicted probabilities for each class.

The AUC score of our AI model for each of the three classes (non-pneumonia, non-COVID-19 pneumonia, and COVID-19 pneumonia) is reported in Table 3. Our AI model achieved higher AUC scores, sensitivity, and specificity for each class on both the internal and external test sets, outperforming the second-best method (CV19-Net) by a large margin. Specifically, our AI model improves the AUC score of the second-best performing algorithms by 1.63% and 3.40% for the test set and the external test set on COVID-19 pneumonia, respectively. Moreover, our AI model improves the sensitivity and specificity of the peer methods by 1.39% and 7.24% for the external test set on COVID-19 pneumonia, respectively. The comparison of ROC curves is presented in Figure 3. Additionally, examples of heatmaps from Grad-CAM [34] and predictions generated by our model are shown in Figure 4. Our proposed model is capable of generating a set of three images for reference, pertaining to a given case of interest, such as JRs. The first image in the sequence corresponds to the original CXR, which is augmented with blue circles indicating the suspicious area showing signs of possible COVID-19 infection, computed by the AI model. The second image displays the heatmaps obtained from the model’s predictions, which are overlaid onto the original CXRs. The regions highlighted by the heatmaps correspond to the anatomical areas that exert the greatest impact on the final model predictions. The final image in the set presents the predicted probabilities for each class, thereby providing an integrated summary of the model’s diagnostic accuracy.

### 3.2. AI-Aided Diagnosis

In order to investigate the extent to which the proposed AI model improves the performance of junior residents (JRs), we conducted a comparison of the performances of the JRs with and without AI assistance, as presented in Table 4. The JRs who participated in this study possessed varying levels of expertise. Specifically, JR1, JR2, and JR3 had approximately 6 months, 1 year, and more than 2 years of experience in interpreting CXRs, respectively. The results indicate that the performance of the junior residents improved in proportion to their level of training. Even without AI assistance, the JR with the most experience (JR3) achieved an AUC score of 0.8657 (95% CI: 0.8633–0.8676), while the JR with the least experience (JR1) attained an AUC score of 0.7813 (95% CI: 0.7785–0.7827). Following AI augmentation, we observed improvements for both JR1 and JR2, achieving AUC scores of 0.8482 (95% CI: 0.8452–0.8511) and 0.8511 (95% CI: 0.8493–0.8526), respectively. Additionally, Table 4 presents Cohen’s kappa score for each JR, with the scores of JR1 (0.5574) and JR2 (0.4651) being smaller than that of JR3 (0.7400), indicating that the AI model had a greater impact on JR1 and JR2 compared to JR3. The detailed performance of all JRs before and after AI assistance is presented in Table 5. From Table 5 we can find that: with the AI model’s assistance, the JR1’s sensitivity has been improved from 0.3889 to 0.6250 on COVID-19 pneumonia diagnosis, the specificity has been improved from 0.7317 to 0.9002 on non-COVID-19 pneumonia, the AUC score has been improved from 0.8121 to 0.8417 and the specificity has been improved from 0.9091 to 0.9339 on non-pneumonia. JR2’s sensitivity has been improved from 0.5000 to 0.5833 on COVID-19 pneumonia diagnosis, the specificity has been improved from 0.8226 to 0.8514 on non-COVID-19 pneumonia, and the specificity has been improved from 0.9008 to 0.9835 on non-pneumonia. Notably, even JR3 with higher training level, the AI model was found to improve sensitivity, from 0.8681 to 0.8902, demonstrating the broad applicability and effectiveness of the model.

## 4. Discussion

Several recent studies have highlighted the potential of chest CT in diagnosing COVID-19 pneumonia, particularly in its early stages [35,36]. However, in healthcare systems with limited resources, CT may not always be available, and CXR is the more commonly used imaging method in clinical practice. Additionally, concerns have been raised by international workgroups regarding the untested specificity of CT in cases where the pre-test probability of COVID-19 infection is low, and CXR is preferred to reduce the risk of nosocomial transmission [37,38,39]. Recent reports have demonstrated that CXR findings correlate well with clinical severity and can be used to predict severe pneumonia [24]. Therefore, the development of AI models that aid in diagnosing COVID-19 pneumonia using CXRs remains an area of active research and is of significant importance.

Junior residents are often the first healthcare professionals to interpret CXRs for suspected COVID-19 patients in clinical practice. However, interpreting CXRs can be challenging, especially when the radiological report may impact patient disposition. This study presents an AI model capable of performing three-class classification for non-pneumonia, non-COVID-19 pneumonia, and COVID-19 pneumonia. The proposed model demonstrates superiority in AUC score, sensitivity, and specificity compared to five peer methods. Although all methods experience performance degradation when tested on the external test set, our model performs better, achieving an AUC score of 0.8594 (95% CI: 0.8594–0.8602), and outperforming the second-best method by a large margin [33].

This study provides compelling evidence for the potential of a trained AI model to enhance the diagnostic performance of junior residents in the field of chest radiography by providing probability outputs and heatmaps. Although previous studies have investigated the augmentation of radiologists’ performance using AI in distinguishing COVID-19 from other types of pneumonia on chest CTs [40], this study specifically focuses on the performance of junior residents, which may have significant implications for both diagnostic imaging and education. The participating JRs possessed varying levels of expertise, with JR1, JR2, and JR3 having approximately 6 months, 1 year, and more than 2 years of experience in interpreting CXRs, respectively. The findings suggest that the performance of the junior residents improved in proportion to their level of training. Furthermore, in addition to the quantitative improvements observed, all three junior residents reported increased confidence when using AI assistance, suggesting that AI may have positive qualitative outcomes as well.

In this study, the adoption of EfficientNet as the backbone for diagnosing COVID-19 pneumonia, and non-COVID-19 pneumonia from CXRs was proposed. Although the model achieved promising performance for the test set and improved decision-making for junior residents, several limitations were noted. Specifically, a significant decrease in performance was observed on the external test set, which is not unique to our method but common in medical image analysis due to domain shift problems [41,42,43]. The training process did not account for differences in data distributions between the TTSH dataset and the external test set, leading to a drop in performance. Medical datasets are often drawn from different domains, even within the same institution, which can pose challenges for deep learning models that assume similar data distributions between training and test sets [43]. Deep learning typically assumes that both the distributions of the training and test sets are similar [44]. If the distributions of both datasets are dissimilar, the performance may drop dramatically. In future work, domain adaption techniques will be explored to align the distributions of different datasets for automated disease diagnoses from CXRs. Furthermore, a purposeful 3-month hiatus was implemented, during which three JRs were tasked with interpreting the same dataset of 500 CXRs with and without AI assistance. Over this three-month period, the diagnostic abilities of the JRs may have improved, potentially influencing the accuracy enhancement achieved when using AI as an assistant.

In summary, our proposed method exhibited a high degree of accuracy in performing a three-class classification of non-pneumonia, non-COVID-19 pneumonia, and COVID-19 pneumonia. Furthermore, the method was observed to improve the diagnostic performance of junior residents in identifying COVID-19 pneumonia on CXRs, a potentially valuable asset in triage and education during the ongoing pandemic.

## 5. Conclusions

This study proposes an AI model that utilizes EfficientNet-b7 [29] as the backbone to differentiate non-pneumonia, non-COVID-19 pneumonia, and COVID-19 pneumonia from CXRs. The training dataset was collected from Tan Tock Seng Hospital and was annotated by experienced senior radiologists. This study showcases the innovative creation of an AI model for three-category CXR classification, emphasizing its ability to enhance the diagnostic precision of less experienced residents. Validated with external data, the model demonstrates practical relevance in real-world scenarios. Upon deployment, the AI model demonstrated its ability to assist junior residents in CXR interpretation and increase their confidence in diagnostic accuracy. Although the proposed AI model improved the performance of junior residents, a performance drop was observed on the external test in comparison to the results on the test set. It indicated that there has been a domain shift between the collected patient dataset (including the training, validation, and test set) and the external dataset. Domain shift is a common issue in practical applications of AI, especially in clinical practice. The clinical practice presents much more heterogeneous acquisition conditions [17] which may lead to the performance of the trained model on external test datasets (or a dataset it encounters when deployed) degrading.

To address the domain gap issue between the training dataset and the external test dataset, we plan to investigate and develop a test-time training domain adaptation model in future work. This model will aim to align the tested CXR in real time with the training data, which is expected to enhance the model’s performance after deployment. By aligning the test CXR with the training dataset, the test-time training domain adaptation model is expected to be more effective and mitigate performance degradation to a large extent. Moreover, it is expected to be more robust to the changing acquisition conditions during CXR screening. 

## Figures and Tables

**Table 1:** Statistics of training, validation, test, and external test sets.

	COVID-19 Pneumonia	Non-COVID-19 Pneumonia	Non-Pneumonia	Total
Training set	425	399	2712	3536
Validation set	61	57	387	505
Test set	121	114	775	1010
External test set	72	49	379	500

**Table 2:** Performance comparison of the proposed model with that of peer methods for the test set and the external test set in terms of AUC scores and 95% confidence interval (95% CI).

	Test Set		External Test Set	
	**AUC**	**95% CI**	**AUC**	**95% CI**
Ahuja’s [30]	0.8982	0.8968–0.8993	0.7680	0.7651–0.7704
nCOVnet [31]	0.8876	0.8854–0.8897	0.6837	0.6012–0.6859
Vaid’s [32]	0.9021	0.8996–0.9038	0.7402	0.7379–0.7425
Apostolopoulos’s [33]	0.9279	0.9229–0.9294	0.8162	0.8145–0.8185
CV19-Net [6]	0.9395	0.9361–0.9407	0.7987	0.7952–0.8032
Ours	0.9520 *	0.9479–0.9585	0.8588 *	0.8570–0.8623

* Denotes statistically significant (*p* >0.05). AUC = the area under the receiver operating characteristic.

**Table 3:** Performance comparison for each class on the test set and the external test set in terms of AUC scores, Sensitivity, and Specificity.

		Test Set			External Test Set		
		**AUC**	**Sensitivity**	**Specificity**	**AUC**	**Sensitivity**	**Specificity**
Ahuja’s [30]	COVID-19 pneumonia	0.9185	0.8966	0.7768	0.7309	0.7361	0.5491
	Non-COVID-19 pneumonia	0.8886	0.8421	0.8136	0.7762	0.7755	0.6408
	Non-pneumonia	0.8964	0.8429	0.8000	0.7740	0.7784	0.6116
nCOVnet [31]	COVID-19 pneumonia	0.8897	0.8793	0.7312	0.6437	0.7083	0.5117
	Non-COVID-19 pneumonia	0.8817	0.8639	0.7739	0.7251	0.7347	0.5322
	Non-pneumonia	0.8882	0.8596	0.7864	0.6860	0.7230	0.5124
Vaid’s [32]	COVID-19 pneumonia	0.9088	0.8448	0.8064	0.7154	0.7500	0.6005
	Non-COVID-19 pneumonia	0.9024	0.8421	0.8500	0.7387	0.6735	0.5854
	Non-pneumonia	0.9010	0.8613	0.8087	0.7451	0.7704	0.5785
Apostolopoulos’s [33]	COVID-19 pneumonia	0.9284	0.8793	0.8497	0.7856	0.7917	0.6519
	Non-COVID-19 pneumonia	0.9234	0.8772	0.8182	0.7938	0.7347	0.7251
	Non-pneumonia	0.9285	0.8586	0.8174	0.8250	0.7863	0.7438
CV19-Net [6]	COVID-19 pneumonia	0.9327	0.8966	0.8360	0.7787	0.8194	0.5958
	Non-COVID-19 pneumonia	0.9565	0.8947	0.8091	0.7882	0.7959	0.5987
	Non-pneumonia	0.9380	0.9241	0.8261	0.8038	0.8470	0.6033
Ours	COVID-19 pneumonia	0.9490	0.9310	0.8519	0.8196	0.8333	0.7243
	Non-COVID-19 pneumonia	0.9541	0.9123	0.8500	0.8348	0.8776	0.7073
	Non-pneumonia	0.9522	0.9338	0.8261	0.8694	0.8918	0.6446

**Table 4:** Performance comparison of JRs and JRs+AI for the external test set in terms of AUC score, 95% confidence interval (95% CI), and Cohen’s kappa score.

Expertise Level	JR1 (∼6 Months)		JR2 (∼1 Year)		JR3 (>2 Year)	
	**w/o AI**	**+AI**	**w/o AI**	**+AI**	**w/o AI**	**+AI**
AUC	0.7813	0.8482 *	0.8214	0.8511 *	0.8657	0.8609
95% CI	0.7785–0.7827	0.8452–0.8511	0.8197–0.8232	0.8493–0.8526	0.8633–0.8676	0.8585–0.8624
Cohen’s kappa score ^1^	0.5574		0.4651		0.7400	

* Denotes statistically significant (*p* >0.05). ^1^ 0.41–0.60 moderate agreement, 0.61–0.80 substantial agreement.

**Table 5:** Performance comparison for each class on the test set and the external test set in terms of AUC scores, Sensitivity, and Specificity.

		JRs			JRs+AI		
		**AUC**	**Sensitivity**	**Specificity**	**AUC**	**Sensitivity**	**Specificity**
JR1 ∼ 6 months	COVID-19 pneumonia	0.6524	0.3889	0.9159	0.7424	0.6250	0.8598
	Non-COVID-19 pneumonia	0.7026	0.6735	0.7317	0.6848	0.4694	0.9002
	Non-pneumonia	0.8121	0.7150	0.9091	0.8878	0.8417	0.9339
JR2 ∼ 1 year	COVID-19 pneumonia	0.7079	0.5000	0.9159	0.7239	0.5833	0.8645
	Non-COVID-19 pneumonia	0.6868	0.5510	0.8226	0.6604	0.4694	0.8514
	Non- pneumonia	0.8581	0.8153	0.9008	0.8981	0.8127	0.9835
JR3 > 2 years	COVID-19 pneumonia	0.7681	0.6250	0.9112	0.7542	0.5972	0.9112
	Non-COVID-19 pneumonia	0.7518	0.6122	0.8914	0.7693	0.5918	0.9468
	Non- pneumonia	0.8968	0.8681	0.9256	0.8902	0.9208	0.8595

## Data Availability

The data are not publicly available due to privacy restrictions.
